# Skarzynski Tinnitus Scale: validation of a brief and robust tool for assessing tinnitus in a clinical population

**DOI:** 10.1186/s40001-018-0347-4

**Published:** 2018-11-01

**Authors:** Henryk Skarżyński, Elżbieta Gos, Danuta Raj-Koziak, Piotr H. Skarżyński

**Affiliations:** 1 World Hearing Center, Institute of Physiology and Pathology of Hearing, 17 Mokra st., Kajetany / 10 Mochnackiego, 02-042 Warsaw, Poland; 2 Heart Failure and Cardiac Rehabilitation Department, Second Faculty, Medical University of Warsaw, Warsaw, Poland; 3 Institute of Sensory Organs, 1 Mokra st., Kajetany, 05-830, Warsaw, Poland

**Keywords:** Tinnitus, Surveys and questionnaires, Validation studies

## Abstract

**Background:**

Many tinnitus scales are available, but all of them have certain limitations. The aim of the current study was to present a psychometric data of a new brief and reliable questionnaire that could be conveniently used for evaluating tinnitus complaint in adults (either with normal or impaired hearing)—Skarzynski Tinnitus Scale (STS).

**Methods:**

The study included 125 participants with at least 1 month of tinnitus duration. All participants were asked to complete the STS, Tinnitus and Hearing Survey (THS), Tinnitus Functional Index (TFI), Tinnitus Handicap Inventory (THI), and Beck Depression Inventory. Psychometric properties of the new tool were tested using exploratory factor analysis (EFA), Pearson bivariate correlation with other tinnitus questionnaires, Pearson bivariate correlation with pure-tone audiometry, Cronbach’s alpha coefficient, limits of agreement, smallest detectable change, and floor and ceiling effects. Norms for tinnitus severity as measured by the STS are proposed.

**Results:**

As a whole, the STS has excellent reliability (ICC = 0.94) and good internal consistency (*α* = 0.91). The results of EFA and content analysis of wording of the items justified the three-factorial structure. The convergent validity was proven by a significant positive correlation with THI, TFI and THS Subscale A scores. Additionally, the authors proposed norms dividing the results into four tinnitus severity grades.

**Conclusions:**

Statistical analysis shows that STS is a brief but robust tool well-suited to clinical practice. A feature of STS is that it takes into account the impact of tinnitus on the patient’s psychological (emotional, cognitive) and functional domains as well as their ability to cope with tinnitus-related distress.

**Electronic supplementary material:**

The online version of this article (10.1186/s40001-018-0347-4) contains supplementary material, which is available to authorized users.

## Background

Tinnitus is an auditory sensation generated by abnormal activation within the auditory system when no external sound is present [1]. It is commonly described by the sufferers as “ringing in the ears”, but it can take many forms, such as buzzing, hissing, chirping, and others. The prevalence of tinnitus is 4.4–15.1% in adults [2] and the number of tinnitus sufferers is significant not only among older adults but also children [3–5].

Tinnitus has a serious impact on everyday life, leading sometimes to poor psychological well-being, insomnia, difficulties in concentration, and others [6–9]. However, tinnitus is difficult to measure objectively because it is almost always a subjective phenomenon, and objective measures of tinnitus such as pitch or loudness only weakly correlate with the impact of tinnitus on various domains of life [8, 10–12]. For this reason, self-reported measures are widely used in clinical practice to quantify tinnitus severity.

Many tinnitus scales are available, but according to systematic reviews not all of them meet the criteria of good measures [13]. In Poland, cross-cultural adaptation and validation have been made for three tinnitus questionnaires: the Tinnitus and Hearing Survey (THS-POL), Tinnitus Handicap Inventory (THI-POL), and Tinnitus Functional Index (TFI-Pl) [14–16]; in the current literature these three have been found to be the most robust among the available tools [17]. However, all these questionnaires also have certain limitations.

The best psychometric properties in several Polish clinical patients were reported to be for the THS; however, its main aim is to differentiate bothersome tinnitus from hearing difficulties, and so its application in tinnitus diagnosis is limited. For the THI, the three-factor structure postulated by Newman et al. [18, 19] has not been confirmed in Polish patients [15], a finding in accordance with other reports [20]. Ultimately, the TFI is the product of a thorough and lengthy development and it is a very valuable assessment tool due to its multidimensionality as well as its ability to diagnose tinnitus in great detail. Notwithstanding, its eight-factor structure has not been fully confirmed [21], and likewise in Polish patients [16]. However, in our clinic many patients point out that some TFI items are not clear (for example, How depressed were you because of your tinnitus) and that the 10-point response scale (choosing one integer from a range 0–10, with definition only for the extreme points) is too wide and difficult to use. Difficulties have also been encountered while assessing tinnitus in patients with hearing problems, which constitute the majority in our tertiary center. From patient reports, it was often almost impossible to distinguish their hearing problems from their tinnitus problems, especially on the Auditory subscale (referring to questions such as ability to hear clearly, understanding people who are talking, or following conversations due to tinnitus) and the Quality of life subscale (questions such as enjoyment of life, social activities, and relationship disturbance due to tinnitus). These questions require a lot of explanation from the examiner, interfering with the study protocol (since the TFI should ideally be a self-reported measure) and this can potentially bias the results.

In clinical practice it was regularly noticed that many patients, despite reporting severe tinnitus, developed strategies to cope with the problem and minimize the impact of tinnitus on their daily life. Since every health complaint can be a source of distress [22], it is worth recording behavioral and cognitive efforts made by patients to manage the difficulties caused by their complaint, in this case tinnitus [23]. However, in the case of tinnitus, the role of particular coping strategies has not yet been firmly established [24]. For example, in a study conducted by Henry and Wilson [25], who compared two groups with self-reported high and low tinnitus distress, no difference was found in terms of coping strategies used by the participants or the benefits derived from the strategies. Additionally, a recent review on coping with tinnitus [26] concluded that although coping is a valuable factor in tinnitus research, there is a lack of specific tinnitus-coping questionnaires which have a solid structure in terms of isolating discrete factors.

Taking into consideration all the above-mentioned limitations of existing research tools, and the importance of evaluating tinnitus-coping strategies during clinical assessment, we decided to develop a new brief but solidly framed questionnaire that could be used in a busy clinic and which was convenient for adult tinnitus patients, either with normal hearing or with hearing impairment, to use. The following assumptions were made while developing the tool: (1) it should measure tinnitus severity and reflect the impact of tinnitus on everyday life; (2) it should be able to assess the efficacy of treatment; (3) it should allow the clinician to quickly and efficiently gauge the general coping difficulties of the patient (who might then be directed to other specialists such as counsellors and psychologists); (4) it should measure only tinnitus, not other hearing problems; (5) it should have appropriate psychometric properties with adequate validity and reliability. Furthermore, in our everyday work we felt the need to have a simple tool that could be used in a busy clinic and which was convenient for adults suffering tinnitus.

### Development of the Skarzynski Tinnitus Scale

An initial draft of the new tool—the Skarzynski Tinnitus Scale (STS, named after the primary author)—consisted of 51 items generated by specialists working with tinnitus patients: a physician, psychologist, hearing aid specialist, and psychometrician. Based on a review of the specialist literature [13, 17, 27, 28], our own clinical experience, and analysis of available tools, the following domains were selected: emotional (negative feelings connected with tinnitus, e.g., anxiety, fear, annoyance), cognitive (intrusive thoughts), functional (impact on everyday life), and coping with tinnitus-related distress (efforts to reduce the negative effects of tinnitus). The domain concerning the impact of tinnitus on hearing was not taken into consideration because of the difficulties described earlier: tinnitus patients often suffer from hearing loss as well and so are unable to specify, for example, whether difficulties in understanding other people’s speech are due to hearing loss or tinnitus.

Seven experts were asked to evaluate the items (physician audiologists, psychologists, an audiophonologist, and hearing aid specialists working with tinnitus patients). They received the set of 51 items with the following instructions: Please assign a score from 1 to 5 to each question based on its usefulness in assessing treatment results in tinnitus patients: 1 for a completely inadequate question, 5 for a fully adequate one.

The criterion for selecting an item was a high average score given by the specialists (above 3.5) and an additional criterion was the absence of any extremely negative scores (completely inadequate). On this basis, the experimental version of the STS consisted of 33 items. A 5-degree scale of answers was established: never, hardly ever, sometimes, often, and always. Some items were reverse-scored to reduce acquiescence bias and extreme response bias. This version was completed by 44 patients and each of them was interviewed about the tool. They were asked if the questions were comprehensible and whether the scale of answers was easy to use. Many patients pointed out that the answers: hardly ever, sometimes, often, etc., were hard to interpret, and several patients found it difficult to understand some questions. Many patients complained about the large number of questions and some claimed to be fed up with them. They suggested the questionnaire should be shorter.

Patients’ opinions were essential. Since it is the patients who complete the questionnaires, our goal was to balance their expectations (concerning comprehensibility, number of items, and convenient way of completing it) with diagnostic requirements. The information acquired during the interviews and the results of the experimental version were instrumental in the final selection of items. The aim was to select up to 20 items with a coherent factor structure as well as good reliability. The most frequently used method to examine dimensionality of data is factor analysis. According to the guidelines given by de Vet [29], items that do not cohere with any other set of factors can be deleted, and Cronbach’s alpha can be used to reduce the number of items while maintaining an acceptable internal consistency. In this study an exploratory factor analysis (EFA) was performed for the set of 33 items to isolate a smaller set of items with a factor structure which would explain over 50% of the variance; at the same time the reliability was checked to ensure that Cronbach’s alpha was above 0.70. According to these criteria, 15 items were finally selected (see Additional file 1: Appendix S1). This final version was the subject of the core analyses presented below.

## Methods

### Participants

The study included 125 participants reporting tinnitus complaints who were consecutive patients attending the Audiology and Phoniatrics Clinic. The main eligibility criteria were age over 18 years, tinnitus of at least 1 month’s duration which lasted more than 5 min at a time, and a lack of mental disorders confirmed in the patient’s medical history. The study was conducted according to the World Medical Association Declaration of Helsinki and was approved by the Ethics Committee of the Institute of Physiology and Pathology of Hearing (Approval Number IFPS: KB/18/2017). Each participant gave written informed consent for participating in the study. Two persons did not complete the STS, so they were excluded from the analysis. Seventy persons completed the STS twice over a period of 3 days during their diagnostic evaluation for tinnitus. No therapeutic procedures were applied during the diagnostic process.

There were 53 women and 70 men in the group. The patients’ ages ranged from 22 to 81 years old (*M* = 50.55; SD = 13.13). Table 1 presents education and place of residence. The period of suffering from tinnitus varied from 1 month to 50 years, with an average of 6.06 years. Most frequently, the tinnitus was bilateral (48%), 38.2% reported unilateral tinnitus (left ear 26%, right ear 12.2%), and 12.2% perceived tinnitus as located in their head (1.6% of patients did not answer the question). In 85.4% of patients, tinnitus was continual, while 12.2% suffered from tinnitus periodically (2.4% missing data).

**Table 1:** Participants’ education and place of residence

	% of participants
Place of residence	
Village	22.8
Town up to 100,000	35.0
Town 100,000–500,000	22.0
Town over 500,000	19.4
Missing data	0.8
Education	
Primary	0.8
Secondary	48.8
Higher	44.7
Missing data	5.7

### Measures

All participants were asked to complete the Skarzynski Tinnitus Scale (STS), Beck Depression Inventory (BDI), Tinnitus and Hearing Survey (THS), Tinnitus Handicap Inventory (THI) and Tinnitus Functional Index (TFI). The authors used their own Polish adaptations of THS [14] and THI [15], the Polish version of TFI (as adapted by Wrzosek et al. [16] and purchased on the basis of an agreement between our institution and Oregon Health & Science University, the questionnaire’s rights holder), and the Polish version of BDI [30–32]. Every patient completed the questionnaires in the same sequence: STS, BDI, THS, THI, TFI. STS was filled first to eliminate the priming effect related to the possible impact of other questionnaires on completing the new tool. In this way, we tried to keep the measurement reliability as high as possible.

#### Tinnitus and Hearing Survey

The Tinnitus and Hearing Survey (THS), developed by Henry et al. [33] is a brief tool to determine how much of a patient’s complaint of tinnitus is due specifically to tinnitus (Subscale A) or to hearing problems (Subscale B). The subscale concerning hyperacusis was not used in the analysis. Although tinnitus and hyperacusis can be related phenomena, this construct was not of interest in the current study.

#### Tinnitus Handicap Inventory

The Tinnitus Handicap Inventory (THI) measures the effects of tinnitus on everyday functioning [18, 19]. Twenty-five items are rated on a 3-point scale (yes, no, sometimes). The higher the score is, the greater the impact on everyday function is. The subscales (functional, emotional, and catastrophic) were used to check the validity of STS.

#### Tinnitus Functional Index

The Tinnitus Functional Index (TFI) [34] provides a composite measure for evaluating the functional impact of tinnitus and takes into consideration a broad range of symptoms associated with tinnitus severity. The questionnaire has eight subscales: intrusiveness, sense of control, cognition, sleep, auditory, relaxation, quality of life, and emotional. Higher scores reflect greater negative impact on everyday functioning. Fackrell et al. [21] showed that TFI is appropriate for measuring intervention-related change.

#### Beck Depression Inventory

Beck Depression Inventory is a self-reporting, 21-item inventory used to assess symptoms of depression [30, 31]. Each of the 21 items is rated on a 0–3 point scale. The global score is the sum of all answers and a higher score indicates greater depressive symptoms.

#### Pure-Tone Audiometry

For all patients, hearing thresholds for air and bone conduction in the right and left ear were determined by an experienced technician at frequencies of 0.125, 0.25, 0.5, 1, 2, 4, and 8 kHz using pure-tone audiometry in a soundproof booth.

### Statistical and psychometric analysis

Construct validity was assessed using EFA. It was performed to test factor structure and to assign items to appropriate factors. The factors were extracted using the principal axis method with oblimin oblique rotation (correlation between factors was assumed). The number of factors was decided by considering the cumulative variance explained (a criterion over 50%), eigenvalues (over 1 for each factor), a screen test, and interpretability. A minimum loading of 0.5 for each item was taken as threshold [29, 35].

Convergent validity was assessed using Pearson bivariate correlations with other tinnitus questionnaires. The predefined criterion for strength of association was a correlation between the global scores of STS and THI, and between STS and TFI, of above 0.7 [36]. At the same time, a correlation between the respective subscales of STS and of the other tools (e.g., functioning subscale of STS or functioning subscale of THI) also needed to be above 0.70.

Discriminant validity was assessed using Pearson bivariate correlations with pure-tone audiometry (PTA) results and THS Hearing. The criteria described by Fackrell et al. [13] were used. Weak (or at most moderate) correlations between STS and PTA and between STS and subscale B of THS were expected because hearing problems should not to be related to tinnitus. Additionally, the groups which, in theory, should differ were compared: it was presumed that patients able to cope with tinnitus distress would have lower scores on THI and TFI than patients who had difficulty coping with tinnitus distress. The hypothesis was tested using a *t* test for paired samples and with a statistical significance threshold *p *< 0.05.

Reproducibility was gauged in terms of internal consistency (Cronbach’s alpha), reliability (intraclass correlation coefficient), and agreement (limits of agreement and the smallest detectable change—SDC). According to the criterion described by Nunnally and Bernstein [35], internal consistency was considered good when Cronbach’s alpha was above 0.70. To measure the reproducibility of STS, intraclass correlation (ICC) was used with a positive rating above 0.70 [36]. Agreement was assessed by calculating the limits of agreement described by Bland and Altman [37] and 95% scores were expected to be within the identified agreement limits. The SDC was defined as a change beyond measurement error (outside the limits of agreement) in stable patients [29].

Responsiveness was assessed in terms of the number of items exhibiting floor and ceiling effects. These effects were considered to be absent if fewer than 15% of the respondents achieved the lowest possible score (for a floor effect) or highest possible score (for a ceiling effect) [36]. For clinical use, norms for severity of tinnitus as measured by STS were proposed. For statistical analysis, IBM SPSS Statistics and AMOS version 24 were used.

## Results

### Descriptive statistics

The analysis began with calculating descriptive statistics (Table 2). The means (*M*) were about 2.0; the highest in the item was concerned with difficulty in sleeping (*M* = 2.43), and the lowest mean in item 9 was concerned with coping with tinnitus by distracting attention (*M* = 1.43). The skewness was lower than 1.0, and kurtosis was generally lower than 1.0 too, but in a few cases it was slightly over 1.0.

**Table 2:** Descriptive statistics of items on the Skarzynski Tinnitus Scale (STS)

Item	Minimum	Maximum	Mean	Standard deviation	Skewness	Kurtosis
STS1	0	4	2.43	1.23	− 0.04	− 0.98
STS2	0	4	2.00	1.18	− 0.03	− 0.99
STS3^a^	0	4	1.84	1.07	0.65	− 0.64
STS4	0	4	2.02	1.31	− 0.08	− 1.18
STS5	0	4	1.63	1.22	0.45	− 0.91
STS6^a^	0	4	1.71	1.16	0.47	− 0.71
STS7	0	4	2.21	1.23	− 0.23	− 1.08
STS8	0	4	2.07	1.15	0.12	− 0.94
STS9^a^	0	4	1.43	0.97	0.81	0.22
STS11	0	4	2.45	1.39	− 0.45	− 1.11
STS12^a^	0	4	2.15	1.11	0.20	− 1.08
STS13	0	4	2.33	1.21	− 0.21	− 1.07
STS14	0	4	1.89	1.31	0.06	− 1.26
STS15	0	4	1.76	1.19	0.29	− 0.86

^a^Items recoded

### Discriminating power of each item

The discriminating power of an item refers to the degree with which it can distinguish between subjects with a high level of a trait and subjects with a low level. This property is related directly to how well the score measures a trait [38, 39]. A corrected item total correlation of more than 0.30 was an acceptable level of item discrimination [35]. Correlations are presented in Table 3. Discriminating power was good for nearly all items, except item 9. This item, concerning coping with tinnitus by distracting attention, not only had the lowest mean but also a very low discriminating power, less than 0.3.

**Table 3:** Corrected item total correlations

Item	Correlations
STS1	0.74
STS2	0.82
STS3^a^	0.50
STS4	0.72
STS5	0.62
STS6^a^	0.42
STS7	0.69
STS8	0.77
STS9^a^	0.07
STS11	0.45
STS12^a^	0.42
STS13	0.66
STS14	0.75
STS15	0.72

^a^Items recoded

### Construct validity

#### Exploratory factor analysis

Exploratory factor analysis was performed to reveal factor structure. The factors were extracted using the principal axis method with oblimin oblique rotation (a correlation between factors was assumed). The number of factors was decided after consideration of the cumulative variance explained, eigenvalues, scree test, and interpretability.

Exploratory factor analysis was performed twice. The first time all items were taken into account. The KMO measure of sampling adequacy was 0.91, and Bartlett’s test of sphericity was significant (*χ*^2^(105) = 987.77; *p *< 0.001). The three-factor solution explained 63.31% of the variance. The first factor explained 46.99% of variance, the second 9.25%, and the third 7.07%. But, this solution was not satisfactory because of item 9, for which communality was very low (0.15) and item 9 did not load on the same factor as other items concerned with coping. Additionally, internal consistency for the potential “coping” factor was lowered by item 9.

In this situation, EFA was performed again, this time with item 9 excluded. Once more, three factors were extracted. The three-factor solution explained 65.9% of variance. The first factor explained 50.28% of variance, the second 8.66%, and the third 6.95%. For two factors, eigenvalues were bigger than 1; for one factor it was 0.97. Table 4 presents factor loadings.

**Table 4:** Factor loadings (pattern matrix)

Item	Factor 1	Factor 2	Factor 3
STS1	*0.448*	0.106	*0.359*
STS2	*0.493*	0.236	0.291
STS3^a^	0.102	*0.664*	0.076
STS4	0.282	0.118	*0.703*
STS5	*0.876*	0.104	0.008
STS6^a^	0.028	*0.452*	0.140
STS7	0.267	0.077	*0.508*
STS8	0.112	0.254	*0.597*
STS10	0.142	0.086	*0.849*
STS11	*0.357*	0.283	0.052
STS12^a^	0.058	*0.581*	0.073
STS13	*0.561*	0.116	0.137
STS14	0.248	0.104	*0.554*
STS15	*0.646*	0.081	0.156

^a^Items recoded

The highest on particular factors kept are in italics

The factorial structure was now clear. Nearly all items were clearly assigned with the exception of item 1, which loaded two factors in a comparable manner. This item concerned emotions (irritation), and it was therefore included in another factor representing items concerned with emotions arising from tinnitus. Table 5 presents correlations between the factors.

**Table 5:** Factor correlation matrix

Factor	2	3
1	0.47	0.60
2		0.57

Factor 1, including items concerning functioning in everyday life, and factor 3, including items concerning emotions and thoughts, were more strongly correlated with each other than with factor 2 concerning coping, and this additionally confirms content validity. The results of EFA and content analysis of wording of the items justified the following assignment:

Psychological distress subscale: items 1, 4, 7, 8, 10, 14;

functional subscale: items 2, 5, 11, 13, 15;

coping subscale: items 3*, 6*, 12* (*items recoded).

### Subscales and global scores

The subscale scores were calculated by summing up the answers to individual questions (0, definitely not; 1, rather not; 2, neither yes nor no; 3, rather yes; 4, definitely yes). The sum was then divided by the maximum score which was theoretically possible to obtain. The resulting scores were on a scale from 0 to 100, where 0 meant no difficulties in a given domain. The total score was calculated by summing up answers from all items and dividing the score by 56 (the maximum possible score). The way of calculating the subscale scores is set out below.

Psychological distress subscale:

((item 1 + item 4 + item 7 + item 8 + item 10 + item 14)/24)*100.

Functional subscale:

((item 2 + item 5 + item 11 + item 13 + item 15)/20)*100.

Coping subscale:

((item 3 + item 6 + item 12)/12)*100. These items, as mentioned before, should be recoded, i.e. 0, definitely yes; 1, rather yes; 2, neither yes nor no; 3, rather not; 4, definitely not.

In Table 6, descriptive statistics of the subscale scores and the global STS score are presented. The mean scores are around 50 points, and patients achieved the highest scores on the Psychological distress subscale, which indicates that the biggest impact of tinnitus is to provoke negative emotions and intrusive thoughts. In Table 7, correlations between scores on the subscales are presented. Correlations between the Psychological distress subscale and the Functional subscale were strong, whereas both subscales were moderately correlated with the Coping subscale. The contribution of the Psychological distress subscale and the Functional subscale to global scores was the highest, while the contribution of the Coping subscale was lower.

**Table 6:** Descriptive statistics of subscale scores and global STS scores

Subscale	Min	Max	*M*	SD	Skewness	Kurtosis
Psychological distress	0	100	53.29	25.57	− 0.06	− 0.98
Functional	0	100	50.85	24.22	− 0.03	− 0.82
Coping	8.33	100	47.49	21.02	0.52	− 0.36
STS global score	3.57	92.86	51.18	21.18	0.01	− 0.85

**Table 7:** Correlations between STS subscales

	Functional	Coping	STS global score
Psychological distress	0.76	0.53	0.94
Functional		0.49	0.90
Coping			0.68

For all correlations *p *< 0.01

### Reproducibility

#### Internal consistency

Internal consistency is a measure of the extent to which items in a questionnaire subscale are homogeneous (i.e., correlated), and so tend to measure the same concept. Cronbach’s alpha was calculated for each subscale of the STS separately and for the total score. For the Psychological distress subscale *α *= 0.91, for the Functional subscale *α* = 0.84, for the Coping subscale *α *= 0.62, and for the STS global *α *= 0.91.

The STS global and Psychological distress subscale had extremely high consistency. The Functional subscale had good internal consistency according to the criterion given by Fackrell et al. [13]. Only the Coping subscale had lower, questionable internal consistency. The internal structure was also analyzed using inter-item correlations (Table 8). All correlations (with the exception of two results for item 5) were statistically significant and positive. Correlations between items forming separate subscales were from moderate to strong.

**Table 8:** Inter-item correlations

Item	STS2	STS3	STS4	STS5	STS6	STS7	STS8	STS10	STS11	STS12	STS13	STS14	STS15
STS1	0.69	0.39	0.61	0.54	0.27	0.70	0.59	0.48	0.34	0.32	0.56	0.65	0.60
STS2	1	0.40	0.65	0.66	0.33	0.57	0.72	0.58	0.41	0.46	0.56	0.61	0.70
STS3^a^		1	0.26	0.31	0.41	0.35	0.44	0.29	0.30	0.36	0.30	0.41	0.30
STS4			1	0.54	0.34	0.59	0.63	0.66	0.31	0.24	0.56	0.65	0.57
STS5				1	0.15	0.49	0.47	0.34	0.35	0.13	0.55	0.47	0.66
STS6^a^					1	0.27	0.34	0.36	0.18^a^	0.28	0.32	0.32	0.35
STS7						1	0.65	0.54	0.33	0.30	0.47	0.61	0.52
STS8							1	0.66	0.33	0.42	0.47	0.68	0.58
STS10								1	0.26	0.34	0.41	0.60	0.41
STS11									1	0.28	0.46	0.35	0.32
STS12^a^										1	0.26	0.27	0.28
STS13											1	0.54	0.58
STS14												1	0.57

*p *< 0.01

^a^Items recoded

#### Reliability

Reliability is a measure of the degree to which subjects can be distinguished from each other based on two testing sessions (test–retest). This property was assessed using intraclass correlation coefficient (ICC), with scores > 0.70 indicating high reliability [36]. ICC was as follows (with 95% CI): for the Psychological distress subscale ICC = 0.93 (0.88–0.96); for the Functional subscale ICC = 0.93 (0.89–0.96); for the Coping subscale ICC = 0.81 (0.69–0.88); and for the STS global ICC = 0.94 (0.90–0.97). As a whole, the STS has excellent reliability similar to Psychological distress and Functional subscales, and the reliability of the Coping subscale was also good.

#### Agreement

Agreement relates to absolute measurement error and indicates which scores, on repeated measurement, are close to each other [36]. Agreement was assessed using two methods: the limits of agreement [37] and the SDC. Limits of agreements were calculated as: *d* ± 1.96* SD_diff_, where *d* is the mean difference between test and retest, and SD_diff_ is the mean difference of the standard deviations.

The SDC was derived from the standard error of measurement (SEM) between two repeated measures: ${\text{SEM }} = {\text{ SD}}_{\text{diff}} /\sqrt 2$, so that ${\text{SDC}} = 1.96*/\sqrt 2 *{\text{SEM}}$ [29, 36]. Table 9 presents data concerning reliability and agreement between two repeated measures. The SDC scores are different than the limits of agreement, because they are based on SEM consistency, not SEM agreement. For the STS global scores, SDC was 18.43 and was higher than the limit of agreement, which was 14.79.

**Table 9:** Reproducibility of STS

Subscale	Mean (± SD)		Reliability		Agreement			
	Test	Retest	ICC (95% CI)	Mean diff	SEM	SDC	Limits of agreement	% of agreement (%)
PD	50.0 (25.58)	45.89 (23.99)	0.93 (0.88–0.96)	4.11	8.84	24.43	20.31–28.53	90.0
F	47.57 (26.71)	44.43 (25.7)	0.93 (0.89–0.96)	3.14	9.35	25.84	22.71–29.9	92.9
C	45.6 (20.84)	42.02 (22.45)	0.81 (0.69–0.88)	3.57	12.26	33.89	30.32–37.47	92.9
STS global	48.19 (21.68)	44.54 (21.48)	0.94 (0.90–0.97)	3.65	6.67	18.43	14.79–22.09	95.8

*PD* psychological distress subscale, *F* functional subscale, *C* coping subscale

**Fig. 1:**
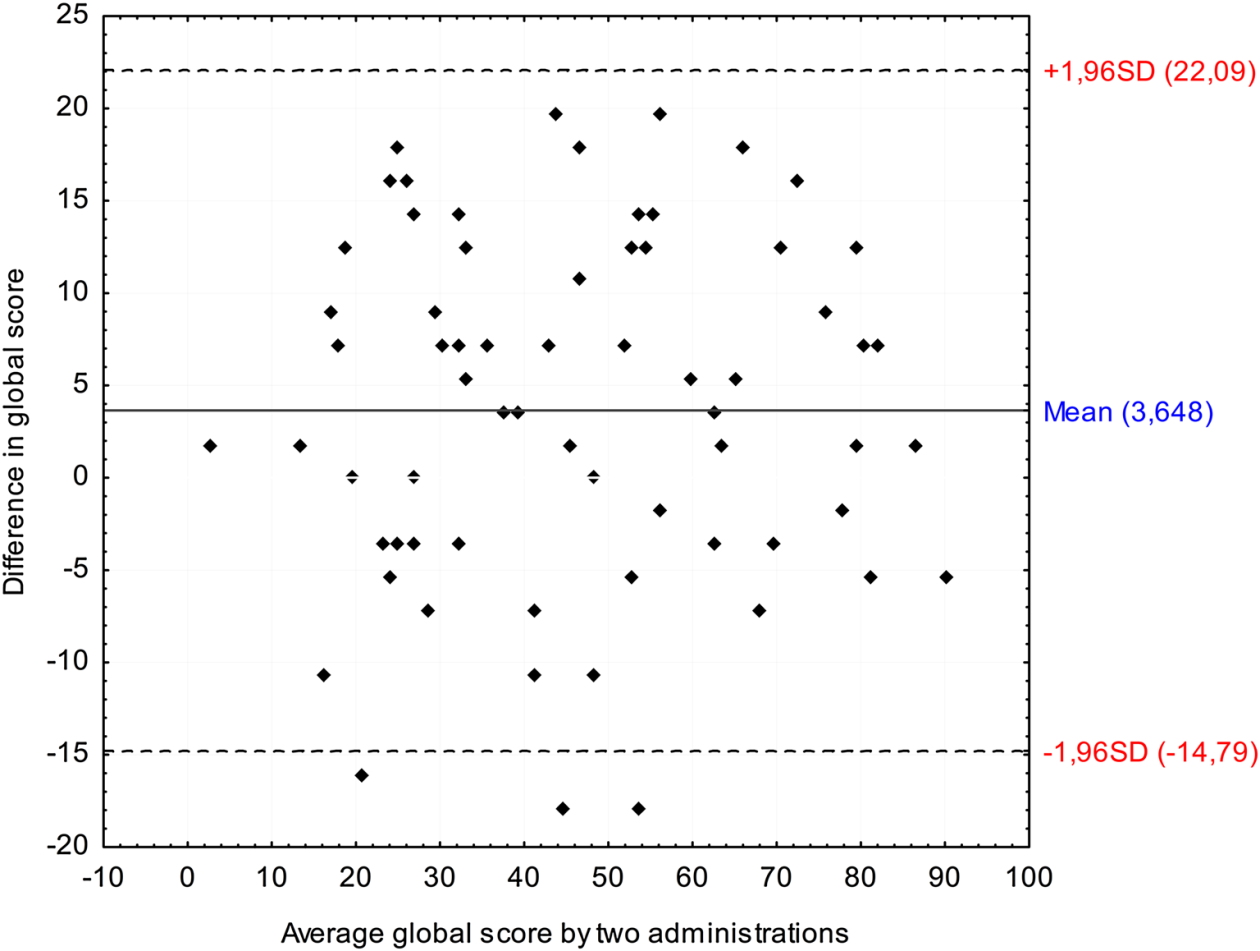
Bland–Altman plot of test–retest agreement of STS global scores

A little under 95% of the scores for individual subscales were within the identified agreement limits. For STS global scores, there was 95% agreement between scores, as shown in Fig. 1.

### Validity

Validity was examined by the degree of correlation with other tinnitus questionnaires and by comparing groups expected to differ due to known characteristics. Convergent validity was assessed as Pearson bivariate correlations. The THS (Subscale Tinnitus), the THI, and the TFI measure a similar construct, so strong—or at least moderate—correlations were expected (Table 10).

**Table 10:** Correlations between STS, THS (Subscale Tinnitus), and THI scores

	Psychological distress	Functional	Coping	STS global score
THS A (Tinnitus)	0.46	0.63	0.24	0.55
THI functional	0.64	0.74	0.42	0.72
THI emotional	0.72	0.60	0.44	0.71
THI catastrophic	0.60	0.57	0.35	0.62
THI global score	0.72	0.71	0.45	0.76

For all correlations ** *p *< 0.01

There was strong and positive correlations between scores on the Psychological distress subscale and the THI Emotional scale, scores on the Functional subscale of STS and the THI Functional scale, and global scores. Also, there was a moderate and positive correlation between THS Hearing and STS global; here, a weaker correlation had been expected because of the low specificity of THS (screening instead of diagnosing specific domains). The weakest correlations occurred between the Coping subscale scores; this is expected since coping is a distinct construct (nevertheless, it should be emphasized that the correlations were significant and positive, and so bigger difficulties in coping with tinnitus were associated with higher THI scores). Table 11 presents correlations between the STS and the TFI scores.

**Table 11:** Correlations between the STS and TFI scores

	Psychological distress	Functional	Coping	STS global score
Intrusiveness	0.49	0.51	0.40	0.55
Sense of control	0.57	0.56	0.40	0.61
Cognition	0.66	0.67	0.37	0.70
Sleep	0.43	0.65	0.39	0.57
Auditory	0.46	0.46	0.36	0.50
Relaxation	0.54	0.69	0.46	0.66
Quality of life	0.57	0.59	0.40	0.62
Emotional	0.71	0.60	0.50	0.72
TFI global score	0.68	0.73	0.50	0.76

For all correlations ** *p *< 0.01

There was strong correlations between scores on the Psychological distress subscale and the TFI Emotional and Cognition scales, and a strong correlation between scores on the Functional subscale STS and the TFI Relaxation and Sleep. There was a moderate correlation between the Coping subscale and the Sense of control subscale, although a stronger correlation was expected. Overall, scores were strongly and positively correlated.

Additionally, validity of the Coping subscale was examined by comparing groups that were expected to differ. The groups were selected on the basis of their median scores on the Coping subscale (Me = 41.67). Patients whose scores were lower than the median were assumed to be coping with tinnitus distress, while those whose scores were higher than the median were assumed to be having difficulties in coping with tinnitus distress. Then, the severity of tinnitus, as measured by the TFI and the THI, was compared (Table 12).

**Table 12:** TFI and THI scores for difficulties in coping with tinnitus distress

	Good coping with tinnitus distress		Bad coping with tinnitus distress		*t* test	*p*
	*M*	SD	*M*	SD		
TFI global score	30.62	19.47	49.46	22.92	4.93	< 0.001
THI global score	33.43	20.97	52.86	25.50	4.63	< 0.001

The differences between subjects with good coping and poor coping with tinnitus distress were statistically significant. In accordance with our presumption, higher tinnitus severity (measured by both TFI and THI) was indicated in patients who had big difficulties in coping with distress due to tinnitus. This shows that the Coping subscale effectively measures a patient’s ability to cope with tinnitus distress. For discriminant validity, the correlations between the STS and the BDI were assessed (Table 13).

**Table 13:** Correlations between STS and BDI scores

	Psychological distress	Functional	Coping	STS global score
BDI	0.50	0.54	0.31	0.55

For all correlations ** *p *< 0.01

Correlations between the STS and the BDI scores were moderate, while for STS coping correlation was weak. This shows that the STS measures a construct which is distinct from depression symptoms. Correlation between the STS global score and the BDI was similar to correlation between the TFI global score and the BDI (*r *= 0.57) reported by Fackrell et al. [21].

Discriminant validity was also examined by looking at correlations between the THS (Hearing subscale) and PTA in terms of average thresholds for the right and left ears (Table 14). There were moderate correlations between STS and THS Hearing. At the same time, there were no correlations between STS and PTA. This shows how difficult it is for patients to distinguish between complaints related to tinnitus and complaints related to hearing loss.

**Table 14:** Correlations between STS and PTA and THS Hearing

	Psychological distress	Functional	Coping	STS global score
Average PTA right ear	0.17	0.13	0.09	0.16
Average PTA left ear	0.04	0.01	0.07	0.04
THS Hearing	0.39**	0.39**	0.26**	0.42

** *p *< 0.01

#### Responsiveness

Responsiveness refers to how well the tool is able to detect major changes and it was assessed in terms of the number of items exhibiting floor or ceiling effects.

A floor or ceiling effect is considered to be present if more than 15% of respondents achieved the lowest or highest possible score [36]. To reveal it, frequency distributions of responses for each of the STS items were examined (Table 15). For the majority of items, the responses were rather uniformly distributed. Only for three items (numbers 4, 5, 14), the fraction of lowest possible responses exceeded 15%. Likewise, only for three items (numbers 1, 11, 13) a ceiling effect might be present, with more than 15% choosing the highest possible response.

**Table 15:** Percentage of responses for the STS items

Item	Responses				
	0	1	2	3	4
STS1	6.5	22.0	14.6	35.8	21.1
STS2	10.6	27.6	22.8	29.3	9.8
STS3^a^	3.3	47.2	22.0	17.9	9.8
STS4	15.4	23.6	18.7	28.5	13.3
STS5	17.1	40.7	13.0	21.1	8.1
STS6^a^	12.2	40.7	20.3	17.9	8.9
STS7	8.9	25.2	16.3	35.0	14.6
STS8	6.5	30.1	26.0	24.4	13.0
STS10	7.3	30.1	14.6	35.0	13.0
STS11	12.2	17.1	13.8	27.6	29.3
STS12^a^	2.4	33.3	24.4	26.0	13.8
STS13	5.7	25.2	17.9	32.5	18.7
STS14	16.3	30.1	13.0	29.3	11.4
STS15	3.8	34.1	22.8	20.3	8.9

^a^Items recoded

**Fig. 2:**
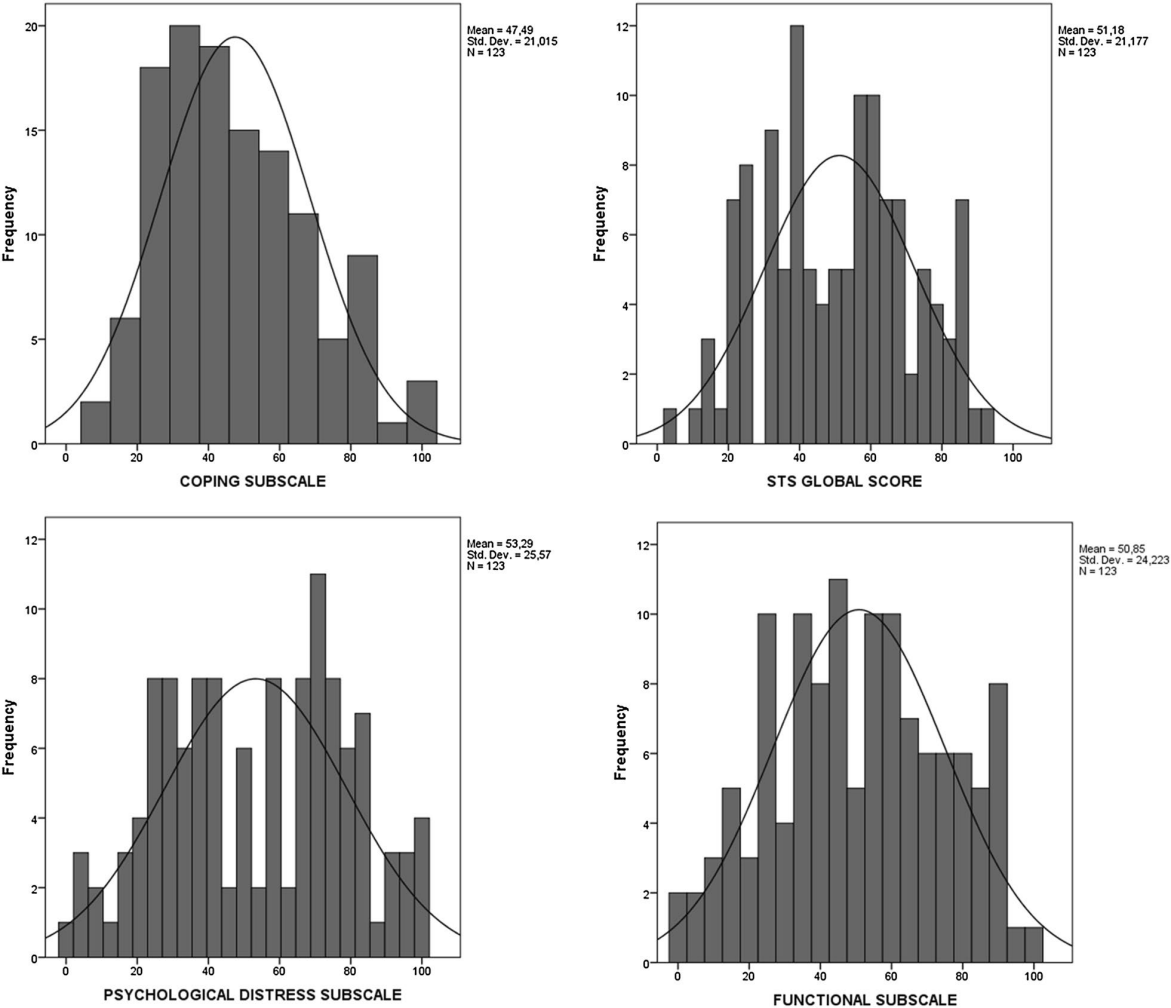
Distributions of STS scores

It also appears that the distribution of scores for subscales and total scores is as significant as the distribution of answers to individual items. Especially in the case of total scores, there is no clear sign of truncated tails (Fig. 2).

### Norms

It is very important, especially in clinical practice, to have the opportunity to assign qualitative meanings to the quantitative scores. Both clinicians and researchers want to identify and quantify the severity of tinnitus. For this reason, an attempt was made to define categories and develop a grading system of STS scores indicating various levels of tinnitus severity.

The distribution of global scores was approximately normal (Kolmogorov–Smirnov test: *Z* (d*f* = 123) = 0.08; *p *> 0.05) with mean *M* = 51.18 and SD = 21.18. Next, the transformation of raw scores into *Z*-scores was made using the formula *Z* = (*X*–*M*)/SD, where *X* is the raw score, *Z* is the standardized score, *M* is the mean, and SD is the standard deviation. In accordance with a normal distribution, the majority of scores (68%) were in the range − 1 to + 1 *Z*-score. Values for − 1 *Z* and + 1 *Z* were treated as the limits of the norm, so that:

$$\left( {X{-} 5 1. 1 8} \right)/ 2 1. 1 8 { } = { 1},{\text{ so}}{:} \, + 1 Z \, = { 72}. 3 6 { } \approx { 72}.$$

$$\left( {X{-} 5 1. 1 8} \right)/ 2 1. 1 8 { } = \, {-} 1,{\text{ so}}{:} \, {-} 1 Z \, = { 3}0.$$

1. Scores below 30 can be considered low and indicate mild tinnitus severity and had a slight impact on everyday functioning.
2. Scores between 30 and 51 can be considered moderate and indicate moderate tinnitus severity and there is a noticeable negative impact of tinnitus on everyday functioning.
3. Scores between 51 and 72 can be considered high and indicate escalated tinnitus severity and there is a considerable negative impact of tinnitus on emotional, cognitive, and functional difficulties. They indicate a poorer ability to cope with tinnitus distress.
4. Scores above 72 (extreme) indicate very high tinnitus severity, a high intensity of negative emotions connected with tinnitus, the occurrence of intrusive thoughts, sleep disturbance, and a handicapped ability to cope with tinnitus distress.

These norms may be very useful in clinical practice to define the current condition of the patient, as a benchmark for treatment progress, and to make empirically based clinical decisions such as starting, continuing, or ending treatment.

## Discussion

The assumption while developing the STS questionnaire was to ensure it would be a convenient tool for patients and, at the same time, would enable physicians to assess the impact of tinnitus on the most important domains of a patient’s functioning. From interviews with patients, we concluded that these domains included emotions, thoughts, and everyday activities. Many patients also emphasized that despite suffering from tinnitus they were able to cope with it.

Factor analysis confirmed the existence of three factors corresponding with the above-mentioned domains. This appears to make sense, since emotions, thoughts, and behaviors constitute a general construct, called attitude by psychologists, whereas the perception of illness and its consequences experienced by the patient affect the way in which the patient copes with their condition.

The participants filled in the STS twice, over a period of just 3 days; such a short time frame results from economic reasons (a 3-day diagnostic hospitalization is done under contract to, and paid by, the Polish National Health Insurance). However, Marx et al. [40] found no difference in the stability of results from a 2-day and 2-week test–retest, so applying a 3-day time frame appears justified in terms other than clinical practicality.

Internal consistency of the whole STS was very high, as was its two subscales of Psychological distress and Functional (unlike the Coping subscale). Although some researchers assume that acceptable internal consistency need only to be higher than 0.6 [41], a 0.7 threshold appears more reasonable. The experience acquired while developing the tool clearly indicates that the issue of coping with tinnitus-related distress is complex and requires a more thorough examination, with bigger contributions from psychologists and therapists. The strategies of coping with stress are varied—from active to passive, from a focus on the problem to a focus on emotions, and so on [25, 42]. This issue should be the subject of separate investigations which might lead to the creation of a separate, specialized tool.

Convergent validity of the STS was verified by its correlation with the most commonly used questionnaires—the THI and the TFI. Significantly, no correlation between the STS scores and pure-tone audiometry scores was found, which confirms that difficulties connected with hearing and with tinnitus are different in nature and that the decision not to include the hearing domain in the tool was correct. At the same time, the Coping subscale proved to be meaningful since, clearly, patients who found it hard to cope with tinnitus-related distress showed higher severity of tinnitus (as measured by the TFI and the THI).

Responsiveness was good. Only on three items a minor floor effect appeared (where a little over 15% of respondents achieved the lowest possible score). On three items, a ceiling effect was visible (over 15% achieved the highest possible score). However, for subscale scores, as well as for global scores, floor and ceiling effects did not appear, and so these scores can be treated as sensitive to change.

A great asset of the STS is the fact that it can be used to detect treatment-related changes. For global scores, the SDC was 18.48, which is a value which can be confidently used to reflect real changes (i.e., not due to measurement error). The norms suggested for the STS may turn out to be very useful in clinical practice. The ability to define a patient’s score as low, moderate, high, or extreme is important and allows a diagnostician or a therapist to decide about the manner and scope of treatment.

A limitation of this study is a slight inconsistency in the content of the Psychological distress subscale. During the development of the STS, its three-factor structure was confirmed. The factor which was later labeled the Psychological distress subscale consisted mostly of emotional items, with two additional cognitive items which seem distinct from the others. However, the authors decided to include these cognitive items in this subscale on the basis of item loadings and intracorrelations between items (0.5 and more), but also according to the findings of psychology which show that negative affect and intrusive thoughts are positively associated [43, 44].

For future research, it would be worth defining a minimal important change (MIC) based not just on the score distribution but on an external criterion (in an anchor-based approach) to check if STS is responsive to treatment-related change. It is also desirable to conduct cross-validation in other groups of patients and test the hypothesis about the three-factor structure of STS. This is especially important to further verify the validity of the STS.

Psychometric validation is a continuous process that requires evaluations in various populations to provide evidence that the measurement tool has the appropriate psychometric properties, adequate validity, and reliability. The Additional file contains not only the Polish version (Additional file 1: Appendix S1) but also the English version (Additional file 2: Appendix S2) of the STS. The English version was created in cooperation between an English translator and the authors of STS, allowing English speakers to acquaint themselves with the content of the questionnaire. We encourage other specialists to apply the STS and optimize its use for research and clinical practice.

## Conclusions

Skarzynski Tinnitus Scale is a brief and robust tool that is very useful for clinical practice. A great advantage of the STS is that it takes into account the impact of tinnitus on both the psychological (emotional, cognitive) and functional domains and the patient’s ability to cope with tinnitus-related distress. The SDC in global score of 18.43 allows it to be used as a measure of treatment-related change. The STS norms convey a clinical meaning to quantitative scores and are easy to apply in clinical practice.

## Additional files

**Additional file 1: Appendix S1.** Skala Szumów Usznych Skarżyńskiego.

**Additional file 2: Appendix S2.** Skarzynski Tinnitus Scale.

## Abbreviations

BDI: Beck Depression Inventory
EFA: exploratory factor analysis
ICC: intraclass correlation coefficient
PTA: pure-tone average
SDC: smallest detectable change
SEM: standard error of measurement
STS: Skarzynski Tinnitus Scale
TFI: Tinnitus Functional Index
THI: Tinnitus Handicap Inventory
THS: Tinnitus and Hearing Survey
