# Supplementary material for: Skarzynski Tinnitus Scale: validation of a brief and robust tool for assessing tinnitus in a clinical population
Source: Eur J Med Res. 2018 Nov 1;23:54. doi: 10.1186/s40001-018-0347-4 (PMC6211414; doi:10.1186/s40001-018-0347-4)
Supplement: Supplementary file 1 — Additional file 1: Appendix S1. Skala Szumów Usznych Skarżyńskiego. [file 40001_2018_347_MOESM1_ESM.pdf]

SKALA SZUMÓW USZNYCH SKARŻYŃSKIEGO

Imię i nazwisko: ………………………………………………………………………………………..

Data wypełnienia: ……………………………… Wiek: …………………… Płeć ☐ K ☐ M

Co to są szumy uszne?

Szumy uszne to różne dźwięki słyszane w jednym uchu, w obojgu uszach lub w głowie i uszach jednocześnie. Dźwięki te mogą przypominać: szum morza, szum wiatru, piszczenie, buczenie, dzwonienie, pukanie, syczenie itp. Są one słyszane tylko przez osobę zgłaszającą ten problem.

Poniżej znajdują się różne stwierdzenia dotyczące szumów usznych. Proszę uważnie przeczytać każde z nich i zastanowić się, czy opisują one Pana/Pani sytuację **w ciągu ostatniego tygodnia.**
Proszę postawić znak **X** w odpowiedniej kratce.

		**Zdecydowanie nie**	**Raczej nie**	**Ani tak, ani nie**	**Raczej tak**	**Zdecydowanie tak**
**1**	Szumy uszne wywoływały u mnie rozdrażnienie.					
**2**	Przez szumy uszne nie mogłem się na niczym skoncentrować.					
**3**	Radziłem sobie z szumami usznymi.					
**4**	Czułem się nieszczęśliwy z powodu szumów usznych.					
**5**	Miałem wrażenie, że przez szumy uszne nie pamiętam o ważnych rzeczach.					
**6**	Przyzwyczaiłem się, że mam szumy uszne.					
**7**	Szumy uszne wywoływały u mnie niepokój.					
**8**	Nie mogłem przestać myśleć o swoich szumach usznych.					
**9**	Odwracałem uwagę od swoich szumów usznych.					
**10**	Zamartwiałem się swoimi szumami usznymi.					
**11**	Miałem trudności ze snem przez szumy uszne.					
**12**	Słyszałem szumy uszne, ale nie zwracałem na nie uwagi.					
**13**	Nie byłem w stanie zrelaksować się z powodu szumów usznych.					
**14**	Szumy uszne wywoływały u mnie złość.					
**15**	Szumy uszne przeszkadzały mi w wykonywaniu codziennych obowiązków.					
