# Supplementary material for: Skarzynski Tinnitus Scale: validation of a brief and robust tool for assessing tinnitus in a clinical population
Source: Eur J Med Res. 2018 Nov 1;23:54. doi: 10.1186/s40001-018-0347-4 (PMC6211414; doi:10.1186/s40001-018-0347-4)
Supplement: Supplementary file 2 — Additional file 2: Appendix S2. Skarzynski Tinnitus Scale. [file 40001_2018_347_MOESM2_ESM.pdf]

SKARZYNSKI TINNITUS SCALE

Name: ……………………………………………………………………………………………..

Date of completing: ……………………………… Age: …………………… Sex □ F □ M

What is tinnitus?

Tinnitus - various sounds heard unilaterally, bilaterally or bilaterally and in the head. The sounds may resemble: the swoosh of the surf, the swoosh of the wind, squeal, buzzing, ringing, knocking, hissing, etc. The sounds are heard only by the person reporting the problem.

Below there are statements concerning tinnitus. Read each statement carefully and decide if it describes your situation **during the last week**. Put X in an appropriate space.

		**Definitely not**	**Rather not**	**Neither yes nor no**	**Rather yes**	**Definitely yes**
**1**	Tinnitus made me irritated.					
**2**	Because of tinnitus I couldn't focus on anything.					
**3**	I coped with tinnitus.					
**4**	I felt unhappy because of tinnitus.					
**5**	I had a feeling that because of tinnitus I don't remember about some important things.					
**6**	I got used to my tinnitus.					
**7**	Tinnitus made me anxious.					
**8**	I couldn't stop thinking about my tinnitus.					
**9**	I distracted my attention from tinnitus					
**10**	I was constantly worried about my tinnitus.					
**11**	I had difficulty sleeping because of tinnitus.					
**12**	I heard tinnitus but I didn't pay attention to it.					
**13**	I couldn't relax because of tinnitus.					
**14**	Tinnitus made me angry.					
**15**	Tinnitus disturbed my everyday duties.					
